# Epigenetic and transcriptional control of adipocyte function by centenarian-associated SIRT6 N308K/A313S mutant

**DOI:** 10.1186/s13148-024-01710-1

**Published:** 2024-07-20

**Authors:** Jan Frohlich, Niccolò Liorni, Manuel Mangoni, Gabriela Lochmanová, Pavlína Pírek, Nikola Kaštánková, Pille Pata, Jan Kucera, George N. Chaldakov, Anton B. Tonchev, Illar Pata, Vera Gorbunova, Eric Leire, Zbyněk Zdráhal, Tommaso Mazza, Manlio Vinciguerra

**Affiliations:** 1 International Clinical Research Center, St. Anne’s University Hospital and Masaryk University, Brno, Czech Republic; 2 IRCCS, Bioinformatics Unit, Casa Sollievo Della Sofferenza, San Giovanni Rotondo, Italy; 3 Mendel Centre for Plant Genomics and Proteomics, Central European Institute of Technology, Masaryk University, Brno, Czech Republic; 4 Laboratory of Functional Genomics and Proteomics, National Centre for Biomolecular Research, Faculty of Science, Masaryk University, Brno, Czech Republic; 5 IVEX Lab, Tallinn, Estonia; 6 RECETOX, Faculty of Science, Masaryk University, Brno, Czech Republic; 7 Department of Physical Activities and Health, Faculty of Sports Studies, Masaryk University, Brno, Czech Republic; 8 Department of Translational Stem Cell Biology, Research Institute of the Medical University, Varna, Bulgaria; 9 Department of Anatomy and Cell Biology, Faculty of Medicine, Varna, Bulgaria; 10 Departments of Biology and Medicine, University of Rochester, Rochester, NY USA; 11 GenFlow Biosciences Srl, Charleroi, Belgium; 12 Clinique 135, Brussels, Belgium; 13 Faculty of Science, Liverpool John Moores University (LJMU), Liverpool, UK

**Keywords:** SIRT6, Epigenetics, Adipogenesis, Histones, Obesity

## Abstract

**Background:**

Obesity is a major health burden. Preadipocytes proliferate and differentiate in mature adipocytes in the adipogenic process, which could be a potential therapeutic approach for obesity. Deficiency of SIRT6, a stress-responsive protein deacetylase and mono-ADP ribosyltransferase enzyme, blocks adipogenesis. Mutants of SIRT6 (N308K/A313S) were recently linked to the in the long lifespan Ashkenazi Jews. In this study, we aimed to clarify how these new centenarian-associated SIRT6 genetic variants affect adipogenesis at the transcriptional and epigenetic level.

**Methods:**

We analyzed the role of SIRT6 wild-type (WT) or SIRT6 centenarian-associated mutant (N308K/A313S) overexpression in adipogenesis, by creating stably transduced preadipocyte cell lines using lentivirus on the 3T3-L1 model. Histone post-translational modifications (PTM: acetylation, methylation) and transcriptomic changes were analyzed by mass spectrometry (LC–MS/MS) and RNA-Seq, respectively, in 3T3-L1 adipocytes. In addition, the adipogenic process and related signaling pathways were investigated by bioinformatics and biochemical approaches.

**Results:**

Overexpression of centenarian-associated SIRT6 mutant increased adipogenic differentiation to a similar extent compared to the WT form. However, it triggered distinct histone PTM profiles in mature adipocytes, with significantly higher acetylation levels, and activated divergent transcriptional programs, including those dependent on signaling related to the sympathetic innervation and to PI3K pathway. 3T3-L1 mature adipocytes overexpressing SIRT6 N308K/A313S displayed increased insulin sensitivity in a neuropeptide Y (NPY)-dependent manner.

**Conclusions:**

SIRT6 N308K/A313S overexpression in mature adipocytes ameliorated glucose sensitivity and impacted sympathetic innervation signaling. These findings highlight the importance of targeting SIRT6 enzymatic activities to regulate the co-morbidities associated with obesity.

**Supplementary Information:**

The online version contains supplementary material available at 10.1186/s13148-024-01710-1.

## Introduction

Obesity is a highly prevalent worldwide: the World Health Organization reports that 39% of the adults are overweight, while 13% is obese [1]. Obesity has almost quadrupled since 1975 [1]. Obesity is a risk factor for metabolic-associated fatty liver disease (MAFLD), hepatic fibrosis, atherosclerosis, hypertension, type 2 diabetes mellitus, metabolic syndrome and respiratory disorders. Moreover, obesity may reduce genomic stability and increase the development of certain cancers [2]. Sirtuins are NAD +- dependent class III histone deacetylases, which modulate numerous cellular functions such as genome stability and nutrient metabolism [3, 4]. In humans there are seven sirtins; SIRT6 is an essential one localized in the nucleus, which has both deacetylase and mono-ADP ribosyltransferase enzymatic activities. SIRT6 was shown to increase life expectancy of rodents, monkeys and humans by increasing genome stability and improving nutrient metabolism [5–9]. Recently, a number of studies have linked SIRT6 enzymatic activities to obesity co-morbidities such as dyslipidemia, MAFLD, diabetes and cardiovascular diseases [9–14]. Interestingly, knock-out (KO) of SIRT6 in the rodent adipose tissue worsens obesity and associated insulin resistance [12, 15], while its transgenic overexpression exerted a protective action against obesity [16].

Obesity-associated aberrant expansion of white adipose tissue (WAT) implicates an augmentation of the existing adipocytes’ size or their quantity thanks to the differentiation of novel adipocytes [17]. Adipocytes derive from pluripotent mesenchymal stem cells (MSCs) becoming committed to differentiate into adipocytes [18]. Recruitment to the adipocyte lineage leads to the production of preadipocytes that in turn differentiate into mature adipocytes. This process, adipogenesis, could be a potential therapeutic approach for obesity. A great number of signaling, epigenetic and transcriptional pathways have been implicated in the commitment of preadipocyte and their differentiation into adipocytes [19]. However, our understanding of the sequential and multifaceted process of WAT adipogenesis as well as the potential of its key modulators for clinical translation is still limited. Chen et al*.* have shown that the depletion of SIRT6 in 3T3-L1 preadipocytes hampers their adipogenesis by enhancing protein kinase CK2 activity [20].

Newly identified genetic/allelic variants of SIRT6 gene have been reported to be linked with healthy aging and human longevity by ameliorating genome maintenance. SIRT6 variants rs183444295 (Ala313Ser → A313S) and rs201141490 (Asn308Lys → N308K), highly incident in Ashkenazi Jews centenarians, appear as promising targets in the enhancement of human lifespan and health span [21]. Ashkenazi Jews longevity is well established, is robustly inherited and correlates with lower incidence of age-related diseases [22, 23]. Although centenarians, in general, have reduced rates of obesity compared to younger age groups [24], Ashkenazi Jews with outstanding longevity are not different with regard to lifestyle factors from the rest of the population, suggesting that genetic/epigenetic factors may be important [25]. SIRT6 variants enriched in centenarians display ADP-ribosylation activity, impinging on genome stability [21]. As mentioned, obesity is a significant risk factor for hepatic fibrogenesis [26], and we recently presented evidence that overexpression of centenarian-associated SIRT6 variants displayed anti-fibrotic impact in 3-dimensional in vitro models [27]. The goal of this study was to investigate the effect of human centenarian-associated SIRT6 variants overexpression in the process of adipogenesis, using a combination of integrated functional/transcriptomics/epigenomics approaches.

## Methods

### Cell culture

3T3-L1 preadipocytes were cultivated and led to differentiation into mature adipocytes following a well-defined protocol, and at the 10th day of differentiation they were used for further analyses [28, 29].

### Lentivirus production and transduction

*Production* self-inactivating lentiviral vectors harboring the wild type and the mutated variants of SIRT6 (a gift of Dr. Laura Sturla, University of Genova, Italy) were built by cloning, using the backbone vector LV2-EF1a_mCer3; UbC_Hygro-WPRE [30]. SIRT6 cDNAs attached to the IRES-driven Katushka2S reporter cassette were positioned under the control of the human EF1α promoter (LV2-EF1a_SIRT6_IRES_Kat2S), which is expressed constitutively. An empty vector without the primary ORF (LV2-EF1a_IRES_Kat2S) was used as a control. To allow the selection of positive cells following transduction, all vectors harbored a separate Hygromycin B resistance cassette, under the control of the UBC promoter. Lentiviruses were assembled by transient transfection of packaging vectors and helper constructs in HEK293T cells [31]. Supernatant containing viral particles was purified from debris by short centrifugation, filtered using 0.45 µm PVDF filter and further concentrated by means of low-speed centrifugation (7000 rpm, 16 h).

*Transduction* confluent cells were cultured into 24-well plate with growth surface area of 2 cm^2^, where 50 × 10^4^ cells were seeded in each well. Upon seeding, cells were transduced with Lentivirus containing SIRT6 constructs: LV2-EMPTY-IresKat2S, LV2-SIRT6(WT)-IresKat2S, LV2-SIRT6(N308K)-IresKat2S and LV2-SIRT6(N308K/A313S)-IresKat2S (Table 1), in basal DMEM media containing 4 µg/mL polybrene transfection reagent (TR-1003, Sigma-Aldrich) with MOI of 1–2. Cells were incubated with viruses for 24 h; subsequently, the fresh medium was added and cells were grown for another 48 h. Then, cells were exposed with selection media including basal DMEM media with 500 µg/mL and hygromycin B (H3274, Merck) for 5 days (the media was changed every other day). Upon selection, surviving cells were grown and controlled for the expression of fluorescent Kat2S signal by fluorescence microscope ex/em 588/635. SIRT6 overexpression was detected by immunoblotting. A list of the cell lines transduced with appropriate LV and their abbreviations is presented in Table 1.

**Table 1:** Lentivirus constructs used

Cell line group	Lentivirus construct	SIRT6 overexpression
CTL	–	–
EMPTY	LV2-EMPTY-IresKat2S	–
WT	LV2-SIRT6(WT)-IresKat2S	Yes
N308K	LV2-SIRT6(N308K)-IresKat2S	Yes
N308K/A313S	LV2-SIRT6(N308K/A313S)-IresKat2S	Yes

### Microscopy imaging

Upon successful differentiation, cells grown on coverslips were rinsed with PBS and fixed with 4% paraformaldehyde at room temperature for 10 min. Following fixation and further washings with PBS, staining with either Oil Red O solution in 40% isopropanol or BODIPY lipid staining dye (1 µg/mL) was performed for 30 min. Coverslips were then processed for microscopy using gelatin (1%) mounting medium containing DAPI (1 µg/mL), and images were detected using an Axio scan Z.1 (Zeiss), with a Hamamatsu ORCA-Flash 4.0 camera, and ImageJ software analysis program (NIH Image, Bethesda, MD) was employed to analyze all immunofluorescence pictures. When cultured in multi-well plates (24 wells), cells stained with ORO or BODIPY were analyzed by spectrophotometer Thermo Fisher Scientific Multiscan GO or by fluorescence measurement by means of Biotek FLX800 gathering adapt fluorescence filters (DAPI 360/460 ex/em; BODIPY 480/520 ex/em), respectively.

### Immunoblotting analyses

Protein extraction and western blotting procedures were carried out as previously described [32–34]. In brief, cells were detached using TrypLE Express, rinsed with 1xPBS and pelleted at 300 g. The recovered pellet was lysed in 1xRIPA lysis buffer supplemented with Halt™ Protease and Phosphatase Inhibitor Cocktail (100X, Thermo Fisher Scientific) at 4 °C for 30 min. Lysates were further centrifuged at 10,000*g* for 10 min at 4 °C, and the protein concentration in the supernatant was assessed by Pierce™ BCA Protein Assay Kit (23225, Thermo Fisher Scientific). Equal amount of protein samples (at least 20 µg) was mixed with 1× Laemmli Sample buffer (1610747, 4x, Bio-Rad), and after boiling at 95 °C for 5 min and cooling on ice, equal volume of protein samples (40 µL) were processed on 10% Mini-PROTEAN® TGX Stain-Free™ Protein Gels (4568034, Bio-Rad) and resolved by electrophoresis running at 120 V for 45 min. Proteins were transferred onto PVDF membranes using Trans-Blot Turbo RTA Mini 0.45 µm LF PVDF Transfer Kit (1704274, Bio-Rad) and Bio-Rad Trans-Blot Turbo Transfer System at 1.3A and 25 V for 10 min. Blocking was performed with 5% bovine serum albumin (BSA, P6154, BioWest) in TBST buffer (20 mM Tris–HCl, pH 7.6, 140 mM NaCl, 0.1% Tween 20) for at least 30 min and exposed to the specific primary antibodies (see below) diluted in TBST blocking solution. Upon washes in TBST buffer, membranes were exposed to secondary antibodies conjugated with horseradish peroxidase diluted, in TBST blocking buffer. Following three further washes with TBST, protein levels were detected by Clarity Western ECL Substrate (1705061, Bio-Rad) and the signal detected on Bio-Rad ChemiDoc XRS+ imaging systems. To quantify protein levels, the scanned membranes were analyzed using the Image Lab™ Software (Bio-Rad).

Here, we used the follow antibodies: Cell Signaling Technology (MA, USA)—rabbit anti Histone H3 (D1H2, 1:1000), rabbit anti-Akt (1:1000), rabbit anti-phospho-Akt (Ser473) (1:1000), rabbit anti-IRS1 (#2382, 1:1000), rabbit anti-IRS2 (#4502, 1:1000), rabbit phospho-IRS-2 (Ser307) (#2381, 1:1000); Abcam (UK)—rabbit anti-SIRT6 antibody (1:1000, EPR18463), secondary goat anti-rabbit IgG HRP-linked (1:2000)—secondary goat anti-mouse IgG HRP-linked (1:2000); Sigma—rabbit anti-phospho-IRS-1 (Tyr612) (I2658, 1:1000), rabbit anti-phospho-IRS-2 (Ser388) (07-1517; 1:1000); Thermo Fisher Scientific—rabbit anti- phospho-IRS2 (Ser1100).

### 2-Deoxy glucose uptake assay

The Glucose Uptake-Glo™ Assay (Promega, UK) was performed on the mature adipocytes. The fully differentiated mature 3T3-L1 adipocytes were cultured in DMEM containing 0.2% BSA for 12 h, and then 50 nM NPY for 12 h and/or 100 nM insulin (INS) for 30 min was added, in order to assess basal glucose consumption. Subsequently, upon discarding the culture medium, the cells were washed with PBS to eliminate the residual glucose. Subsequently, 3T3-L1 adipocytes cells were incubated with 1 mM 2-deoxyglucose (2-DG) for 10 min and further processed according to the manufacturer’s instructions. Following a brief incubation step, an acid detergent solution (stop buffer) was added. Finally, a neutralization buffer was added to the acid, followed by a detection reagent. Recombinant NPY was purchased from Sigma-Aldrich (UK).

### qRT-PCR

Total RNA was obtained from undifferentiated and mature 3T3-L1 cells with TRIzol Reagent (Invitrogen, CA, USA) and using column separation ( RNeasy Mini Kit, Qiagen, Germany). DNASEI was employed to avoid DNA contamination. The integrity of RNA was determined using Agilent RNA 6000 Nano Kit, Agilent 2100 Bioanalyzer (both Agilent Technologies, CA, US) and TapeStation (Agilent Technologies). For RT-PCR, 1 µg of total RNA was employed to synthesize cDNA by means of a High-Capacity cDNA Reverse Transcription Kit (Thermo Fisher Scientific, MA, US). RT-PCR was performed using StepOnePlus™ Real-Time PCR System (Applied Biosystems, Darmstadt, Germany) and SYBR™ Select Master Mix (Thermo Fisher Scientific, MA, USA). The sequences of the mouse primers employed in this work are listed in Supplemental Table 1.

### RNA-Sequencing (RNA-Seq)

250 ng of purified RNA was used to prepare indexed libraries, with a NEB Next Ultra II Directional RNA Library Prep Kit with polyA selection (Illumina, UK). The libraries were pooled in equimolar amounts, processed for cluster generation and sequenced on an Illumina MiSeq System (Illumina) in a 1 × 75 format or Illumina NovaSeq sequencer (run length 1 × 161 nt). FastQC was used for checking the quality of raw paired-end fastq reads [35]. Trimmomatic v0.36 [36] with settings CROP:250 LEADING:3 TRAILING:3 SLIDINGWINDOW:4:5 MINLEN:35 and an adapter file containing (AGATCGGAAGA) were used for the adapters and quality trimming of raw fastq reads. Trimmed RNA-Seq reads were mapped against the mouse genome (mm38) and Ensembl GRCm38 v.93 annotation using STAR v2.7.3a [37] as splice-aware short read aligner and default parameters except -outFilterMismatchNoverLmax 0.1 and -twopassMode Basic. Several tools such as RSeQC v2.6.2 [38], Picard toolkit v2.18.27 [39], Qualimap v.2.2.2 [40] and BioBloom tools v 2.3.4-6-g433f [41] were used of the quality control after alignment concerning the number and percentage of uniquely- and multi-mapped reads, rRNA contamination, mapped regions, read coverage distribution, strand specificity, gene biotypes and PCR duplication. The differential gene expression analysis was determined by means of RSEM tool v1.3.1 [42] and further processed with the Bioconductor package DESeq2 v1.20.0 [43]. Data generated by DESeq2 with independent filtering were selected for the differential gene expression analysis because of its conservative features and to discard potential false positive results. Genes were retained as differentially expressed based on a cut-off of adjusted *p* value ≤ 0.05 and log2(fold change) ≥ 1 or ≤ − 1. Clustered heatmaps were produced using R package pheatmap v1.0.10 [44], and volcano plots and MA plots were generated using ggplot v3.3.3 package [45] and ggpubr v0.4.0 package, respectively [46]. Ingenuity Pathway Analysis (IPA, spring 2018 release, QIAGEN Inc., https://www.qiagenbioinformatics.com/products/ingenuity-pathway-analysis) software package was employed for the functional and pathway enrichment analyses on differentially expressed genes. We considered genes as differentially expressed between groups if their expression values significantly differed by  > twofold with a *p* ≤ 0.05. Correlations were generated by Pearson Chi-square test for categorical variables (variables with limited or fixed, number of possible values).

### Histone post-translational modifications assessment by mass spectrometry

#### Histone extraction

Histone extraction protocol was described in our previous work [47]. At least three replicates of each sample were carried out.

#### Chemical derivatization of histone extract

The volume of histone extracts was reduced in vacuum concentrator to 5 μL, 5 μL of acetonitrile (ACN; Honeywell, USA) was added, and the samples were subjected to microwave-assisted histone derivatization using trimethylacetic anhydride (Merck Millipore, Burlington, MA, USA) according to a previously published procedure [48].

#### LC–MS/MS and database search of histone peptides

Chemically derivatized peptides were analyzed with a LC–MS/MS made of an Ultimate 3000 RSLC-nano system connected to an Orbitrap Lumos Tribrid spectrometer (Thermo Fisher Scientific) equipped with a Digital PicoView 550 ion source (New Objective) and Active Background Ion Reduction Device (ESI Source Solutions) [48].

#### Evaluation of mass spectrometric data

Proteome Discoverer software (Thermo Fisher Scientific; version 2.2.0.388) with in-house Mascot search engine (Matrix Science, version 2.6.2) was used to analyze the raw mass spectrometric data files and to compare acquired spectra with entries in the UniProtKB human database (version 2021_12; 20594 protein sequences), cRAP contaminant database (downloaded from http://www.thegpm.org/crap/) and in-house histone human database (version 2019_10; 52 protein sequences). Mass tolerances for peptides and MS/MS fragments were 10 ppm and 0.03 Da (0.5 Da for cRAP), respectively. We set semi-Arg-C for enzyme specificity allowing up to two missed cleavages. For inquires against cRAP database, the variable modification settings were trimethylacetylation (K, N-term, S, T, Y), acetylation (K), deamidation (N, Q) and oxidation (M). For histone database searches, they were acetylation (K), methylation (K, R), dimethylation (K), trimethylation (K), phosphorylation (S, T) and trimethylacetylation (K, N-term, S, T, Y), and for inquiries against UniProtKB human databases, it was trimethylacetylation (K, N-term, S, T, Y). We manually verified selected histone peptide identifications and we quantified them from the peak areas derived from the EICs using Skyline (64-bit, v. 23.1.1.268 software), which included identification alignment across the raw files based on retention time and m/z.

The relative abundances of histone peptides were determined using R script in KNIME Analytics Platform, as we previously described [49]. The data analysis was carried out in R version 3.6.3 (https://www.R-project.org/) using the compositions and Hotelling R packages for ilr and alr transformations, and Hotelling T2 test, respectively (https://CRAN.R-project.org/package=compositions). Hotelling: Hotelling’s T^2 Test and Variants. R package version 1.0–5. https://CRAN.R-project.org/package=Hotelling).

#### Chromatin immunoprecipitation-PCR (ChIP-PCR)

Chromatin immunoprecipitation was performed in AdiE, AdiWT and AdiCent cell lines with a previously described protocol [29, 50, 51], using antibodies to acetylated H3K9/K14 (ab232952 Abcam, 1:1000 dilution) and IgG (ab150081, Abcam, 1:1000 dilution). At the end of the procedure, proteins were digested with proteinase K, and the remaining DNA was purified using a QIAquick PCR Purification Kit (QIAGEN) and subjected to qPCR with Irs1- (sense, 5′- GGAGGCGGGCTGCCAAGTCC -3′; antisense, 5′- TGGTGGCGGCGGGGACTGTT-3′), Irs2- (sense, 5′- AAGCACAAGTACCTGAT -3′; antisense, 5′- GCGGTACCAGCCCTCC-3′) or Pik3ca- (sense, 5′- AGAAGAACGCACAGCAACG -3′; antisense, 5′- TTACACCCATAGAGGAAACGA 3′) specific primers; or with primers against LINE-1/ORF1 (sense, 5’ –TGGAAGAGAGAATCTCAGGTGC-3’; antisense, 5’-TTGTGCCGATGTTCTCTATGG-3’).

### Statistical analyses

GraphPad Prism software (version 7.00 for Windows; GraphPad Inc., CA, USA) was used for all statistical analyses. To statistically compare groups, we used the parametric Student’s *t* test, if the data had normal distribution in all tested subgroups; if they were not we used the non-parametric Mann–Whitney *U* test instead. To assess statistical significance between more than two groups, a parametric One-Way ANOVA was employed when the data had a normal distribution, or otherwise a non-parametric Kruskal–Wallis test. Independent experiments were performed out at least 3 times with 3 technical replicates. The data are represented as the means ± SD (unless indicated otherwise). Differences were considered statistically significant at *p* < 0.05 (*), *p* < 0.01 (**) and *p* < 0.001 (***).

## Results

### SIRT6 overexpression, wild type or mutant (N308K/A313S), increases adipogenic differentiation in 3T3-L1 cells

**Fig. 1:**
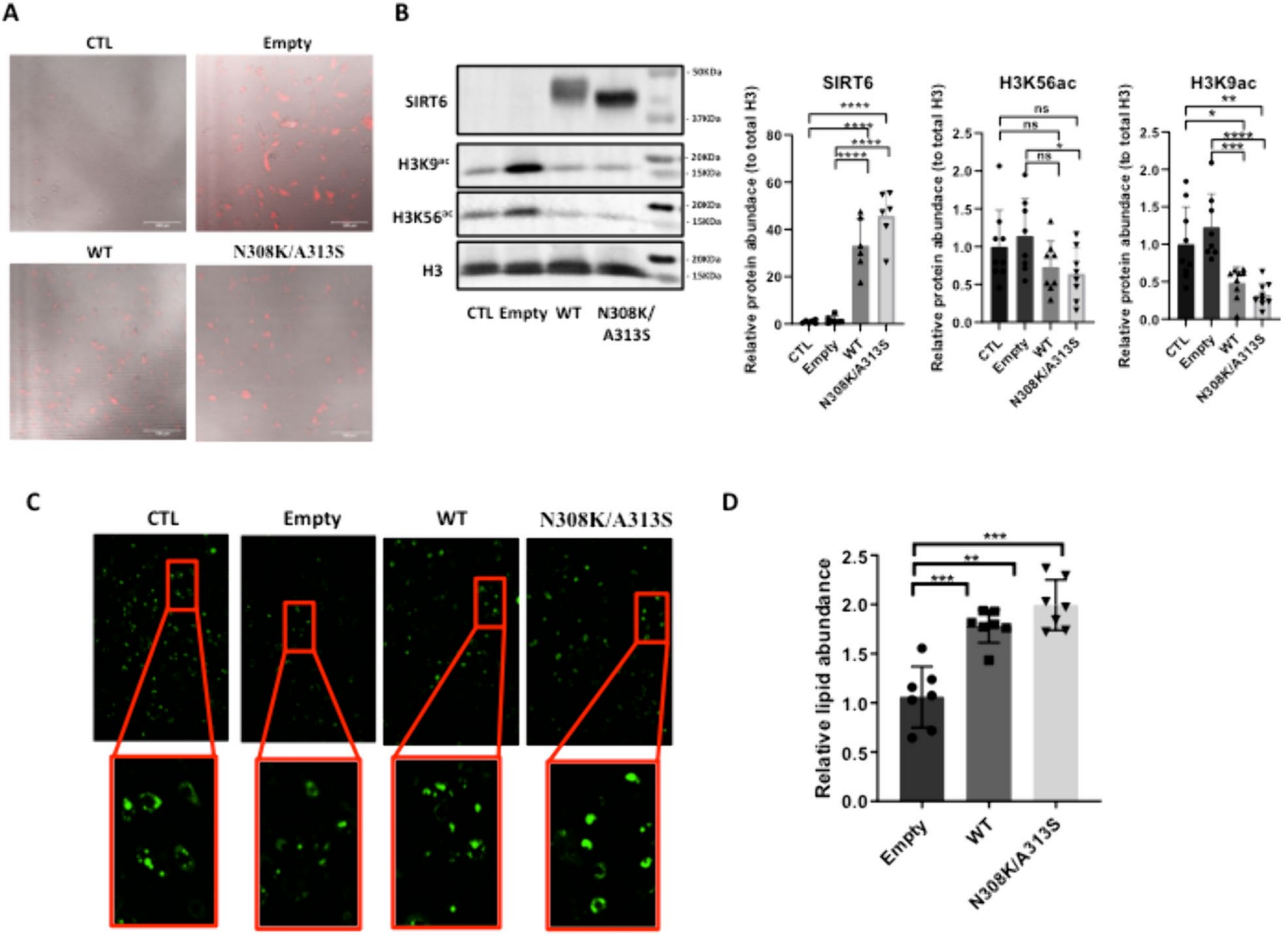
LV-mediated SIRT6 mutant overexpression and its impact on adipogenesis 3T3-L1 cells. **A** Far-red fluorescence protein Katushka2S contained in the LV cassette, alone in the empty group, or together with one of SIRT6 versions (WT, N308K/A313S), demonstrating the successful infection. **B** Immunoblotting to assess SIRT6 protein expression and acetylation levels of SIRT6 target H3K9 and H3K56 sites, in the groups infected with LV-SIRT6 (WT or N308K/A318S) compared to either empty or control (CTL) cells (*left panel*); densitometric quantification as in (**B**) (*right panel*). **C** Intracellular lipid levels quantification of BODIPY measurement in 3T3‐L1 preadipocytes, infected with empty LV or LV to overexpress SIRT6 WT or N308K/A318S mutant and then undergoing adipogenic differentiation. **D** ImageJ-assisted quantification of relative lipid abundance as in (**C**). *p < 0.05; **p < 0.01; ***p < 0.001 (Mann–Whitney *U* test, compared with CTL samples)

It was previously shown that lack of SIRT6 in 3T3-L1 preadipocytes hampers their adipogenesis [20]. To analyze the role of SIRT6 wild-type (WT) or SIRT6 centenarian-associated mutant (N308K/A313S) overexpression (OE) in adipogenesis, we created stably transduced preadipocyte cell lines using lentivirus on 3T3-L1 preadipocytes. The signal of the far-red fluorescence protein Katushka2S contained in the LV cassette, alone in the empty group, or together with one of SIRT6 versions (WT, N308K/A313S) was detectable, proving the successful infection (Fig. 1A). 3T3-L1 control (CTL) cells did not express Katushka2S. SIRT6 protein expression was detected by immunoblotting (Fig. 1B), corroborating the robust increase in SIRT6 levels in the conditions transduced with LV-SIRT6 compared to either empty or CTL cells. One of the functions of SIRT6 is to repress gene expression by removing acetylation of H3K9, H3K18 and H3K56 sites at gene promoters [52–54]. Consistently, in cultured 3T3-L1 cells overexpressing SIRT6 the amounts of acetylated histone H3K9 were significantly diminished, while H3K56Ac displayed a decreased trend (Fig. 1B). Hence, together with the increased SIRT6 expression, there was an augmentation in its deacetylase activity. In order to examine the effect of SIRT6 OE on adipogenic differentiation, we determined the degree of differentiation using BODIPY lipid staining at the end of the differentiation protocol. OE of SIRT6 WT or SIRT6 N308K/A313S significantly increased lipid accumulation, as assessed by quantitative photometric and microscopic analyses of BODIPY staining (Fig. 1C, D).

### Overexpression of SIRT6 wild type or centenarian-associated (N308K/A313S) trigger distinct histone post-translational modifications (PTM) profiles in mature adipocytes

**Fig. 2:**
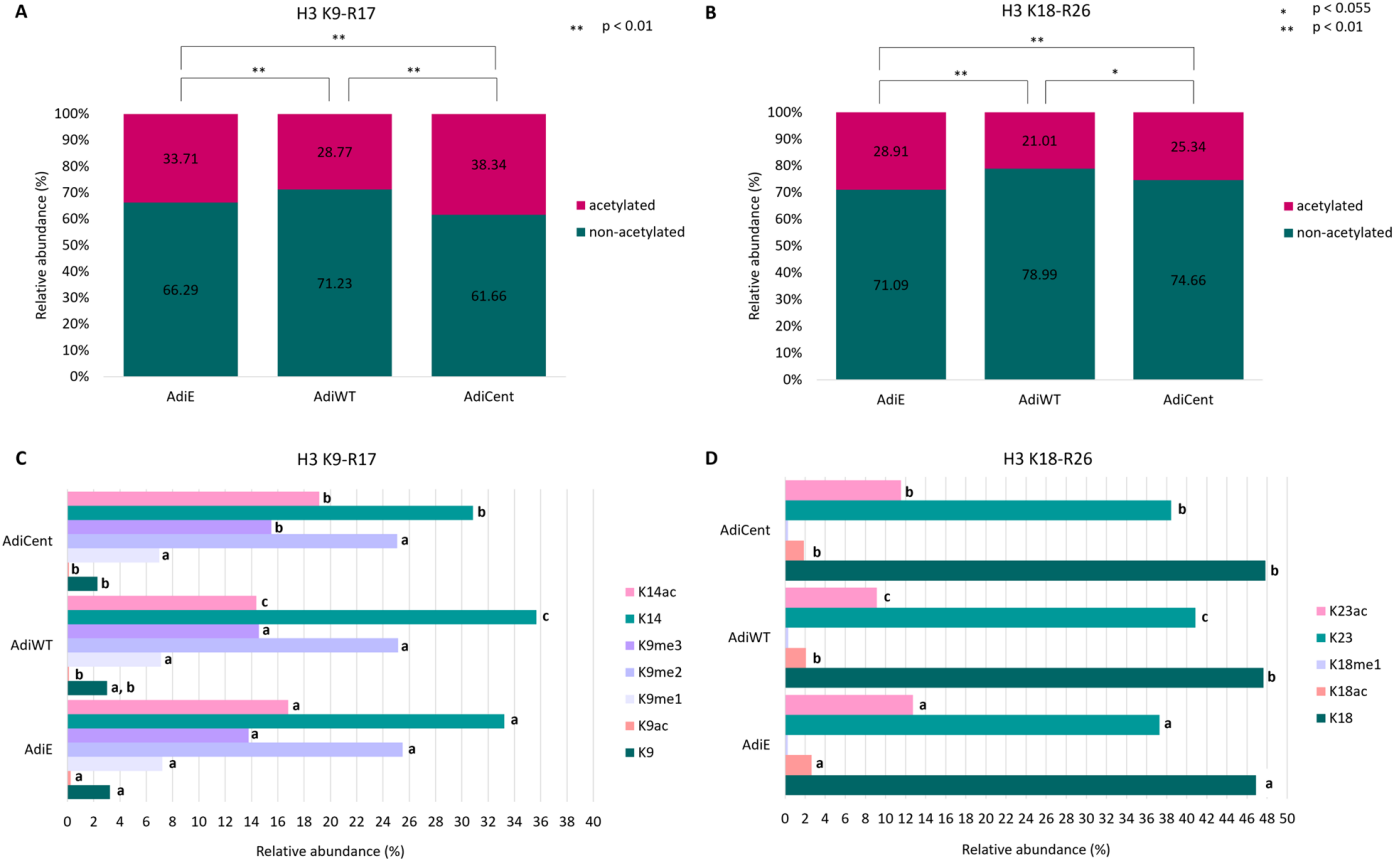
The impact of SIRT6 OE on H3 acetylation profiles. Differences in global acetylation of **A** H3K9STGGKAPR17 (H3K9 − R17) and **B** H3K18QLATKAAR26 (H3K18 − R26) peptides in control AdiE, and SIRT6 OE-samples (AdiWT and AdiCent) with a detailed view **C** on the levels of identified histone marks. Numbers correspond to the median values (*n* = 3–4) of precursor peak areas in percentages. For each histone mark, different letters indicate significant differences between AdiE, AdiWT and AdiCent according to the Student *t* test at *p* < 0.055

**Fig. 3:**
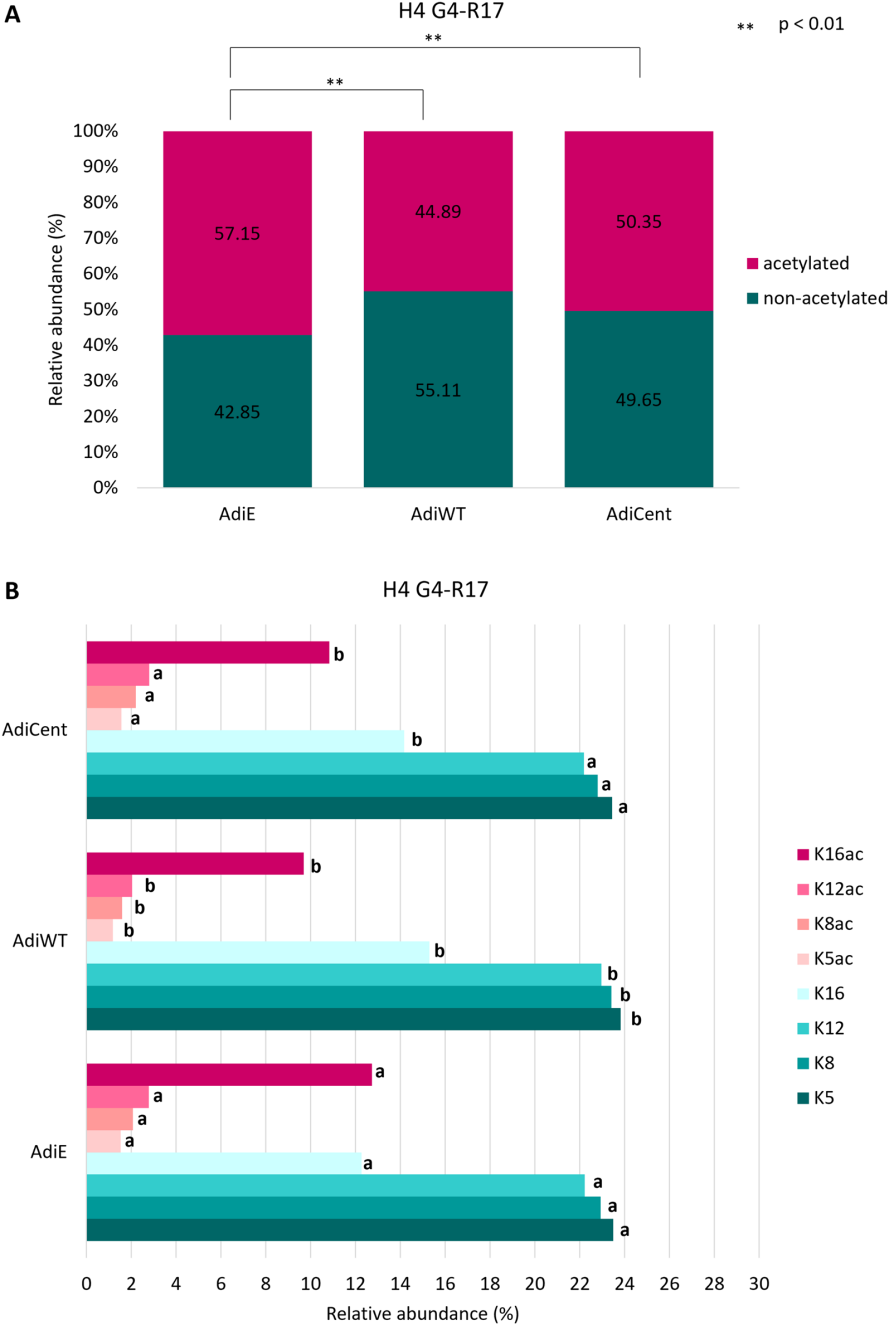
The impact of SIRT6 OE on H4 acetylation profile. Differences in **A** global acetylation of H4G4KGGKGLGKGGAKR17 (H4G4 − R17) peptides in control AdiE, and SIRT6 OE-samples (AdiWT and AdiCent) with a detailed view **B** on the levels of identified histone marks. Numbers correspond to the median values (*n* = 3–4) of precursor peak areas in percentages. For each histone mark, different letters indicate significant differences between AdiE, AdiWT and AdiCent according to the Student *t* test at *p* < 0.055

Of all histone post-translational modification (PTM) types, acetylation and methylation are the two most well-studied types, and they functionally interact to fine-tune transcriptional outputs [55]. To gain insight into the SIRT6-dependent epigenomic regulation during adipogenesis, we performed a comprehensive analysis of histone acetylation/methylation PTMs by mass spectrometry (LC–MS/MS) in 3T3-L1 differentiated adipocytes (AdiE, AdiWT and AdiCent). H3.1, H3.3 and H4 proteins were identified with high sequence coverage, including peptides that carry PTMs (Figure S1). The peptides with identical amino acid sequences in H3.1 and H3.3, i.e., H3K9STGGKAPR17 (H3K9−R17), H3K18QLATKAAR26 (H3K18−R26), H3Y54QKSTELLIR63 (H3Y54−R63) and H3E73IAQDFKTDLR83 (H3E73−R83), were quantified together for both variants. On the other hand, H3K27−R40 peptides which are represented by unique sequences for each H3 variant, i.e., H3.1K27SAPATGGVKKPHR40 (H3.1K27−R40) and H3.3K27SAPSTGGVKKPHR40 (H3.3K27−R40), were quantified separately. From the overall levels of unique peptides, we determined that H3.1 is a highly predominant variant in differentiated adipocytes. The percentage of H3.1 and H3.3 corresponded to 94% and 6%, respectively, meaning that global PTM levels are primarily driven by the H3.1 modification status. Major changes between samples were found in acetylation levels of H3K9−R17, H3K18−R26 and H4G4KGGKGLGKGGAKR17 (H4G4−R17) peptides (Figs. 2 and 3). Those peptides had significantly lower overall acetylation state in AdiWT compared to AdiE (*p* < 0.01; Figs. 2A, B and 3A). The deacetylase activity of centenarian-associated SIRT6 mutant in AdiCent was weaker than activity of SIRT6 in AdiWT, leading to a significantly higher acetylation level in AdiCent compared to AdiWT. However, except for H3K9−R17, significantly lower levels of acetylated peptides were still observed when compared AdiCent with AdiE (*p* < 0.01). While wild-type SIRT6 OE led to a significant decrease of H3K14ac, this histone mark remained at high levels in AdiCent, even higher levels than in AdiE (Fig. 2C). In addition, a slightly higher level of H3K9me3 was found in AdiCent compared to both AdiWT and AdiE. Low abundant H3K9ac, as well as H3K18ac and H3K23ac marks (Fig. 2C, D), had lower levels in AdiWT and AdiCent compared to AdiE. Further, we observed a distinct impact of mutant and wild-type SIRT6 on the acetylation status of histone H4. In AdiWT, lower levels of all acetylated lysines accompanied by higher levels of non-acetylated counterparts compared to AdiE were found. On the contrary, mutant SIRT6 targeted H4K16ac only (Fig. 3B).

**Fig. 4:**
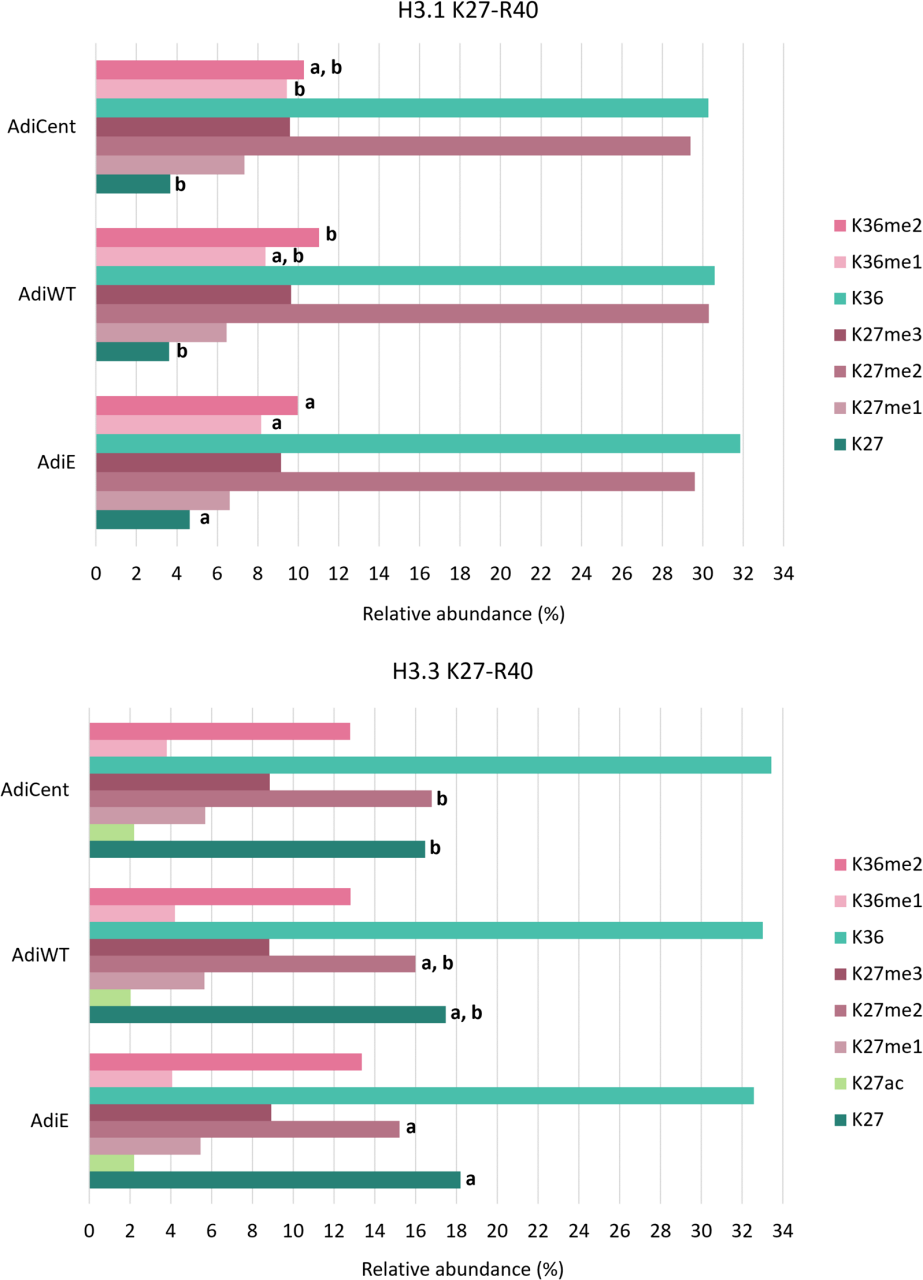
The impact of SIRT6 OE on K27–R40 methylation profiles in H3.1 and H3.3 variants. Numbers correspond to the median values (*n* = 3–4) of precursor peak areas in percentages. For each histone mark, different letters indicate significant differences between AdiE, AdiWT and AdiCent according to the Student t test at *p* < 0.055

Regarding methylation profile, slightly increased H3.1K36me1 and /or me2 levels were found in OE-samples compared to AdiE (Fig. 4). In AdiCent, a higher level of H3.3K27me2 was observed.

### Overexpression of wild type or centenarian-associated (N308K/A313S) SIRT6 activate divergent transcriptional programs during adipocyte differentiation

**Fig. 5:**
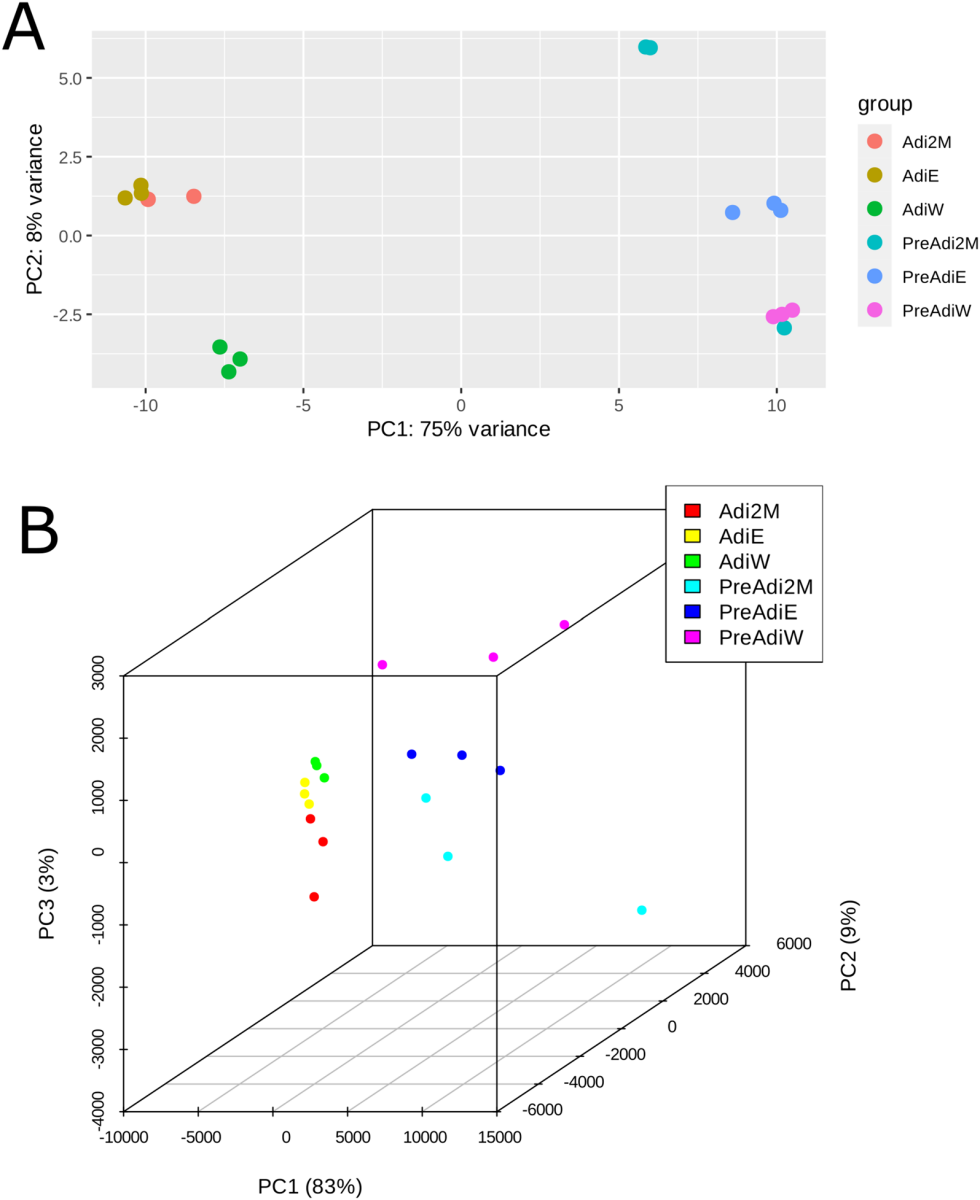
**A** 2D and **B** 3D visualization of the principal component analysis (PCA) performed on the variance-stabilized counts obtained with DESeq2. The PCA allows the visualization of the overall gene expression structure across samples, identifying any potential sources of variation or clustering in the data

**Fig. 6:**
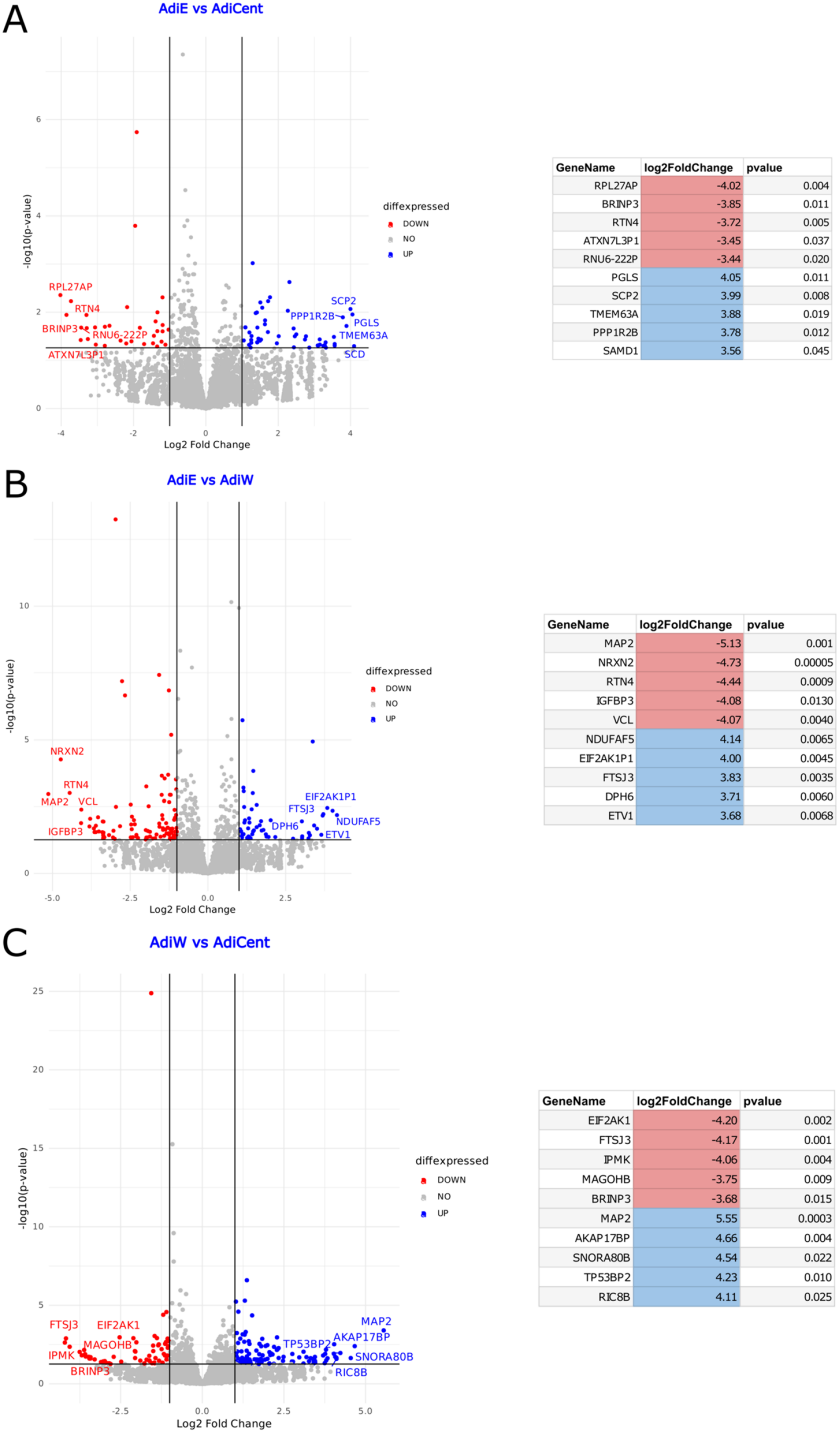
Volcano Plot visualization of Differentially Expressed Genes (DEGs) between adipocytes. The x-axis represents the log2 fold change (log2FC), and the y-axis represents the -log10 of the p value. Genes with significant differential expression are highlighted in blue (up-regulated genes) and red (down-regulated) and are reported in the flanking table with the same color code. **A** AdiE vs. AdiCent. **B** AdiE vs. AdiWT. **C** AdiWT vs. AdiCent

**Fig. 7:**
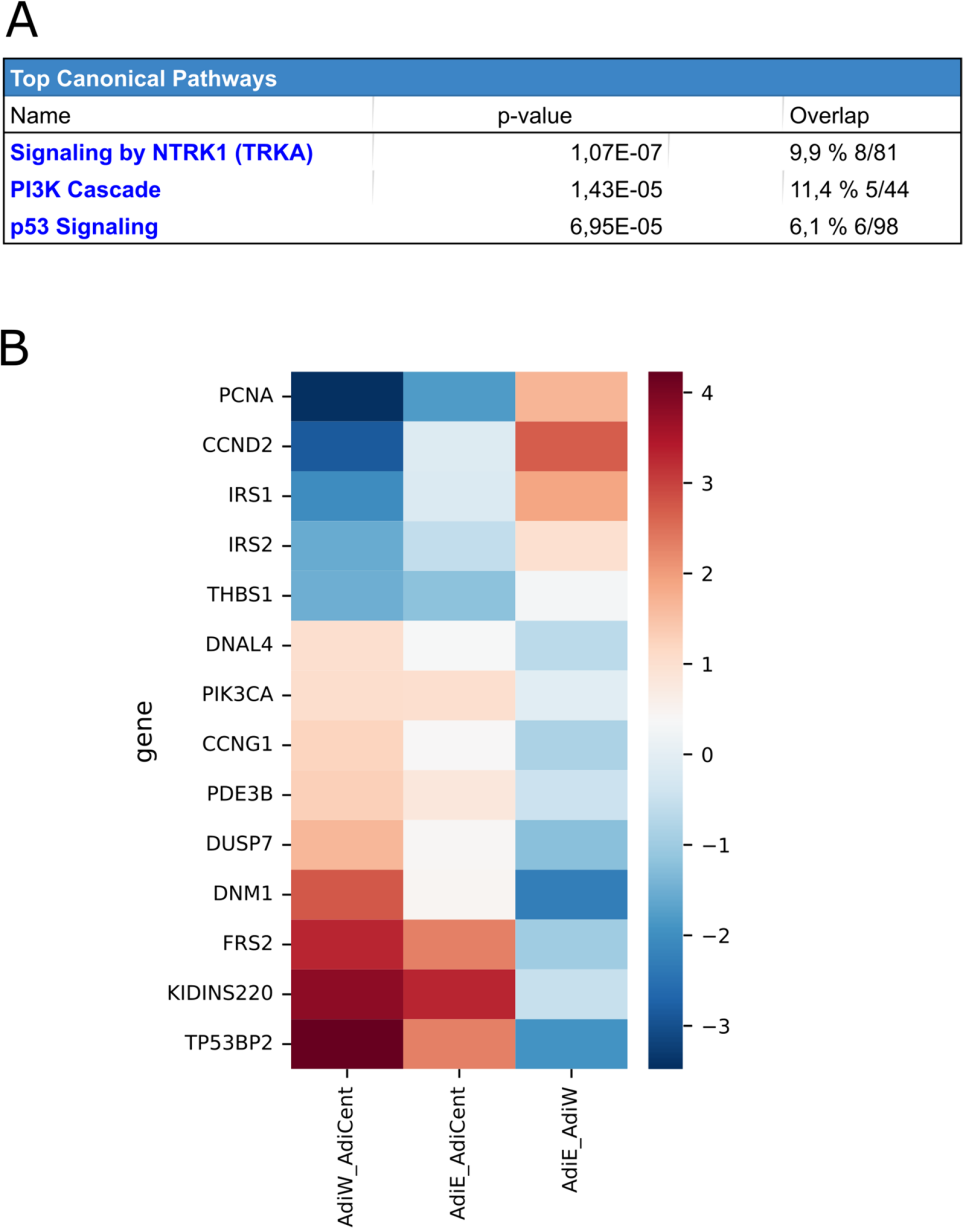
Bioinformatic analysis of RNA-seq data. **A** Top 3 Canonical Pathways obtained with an Ingenuity Pathway Analysis (IPA) enrichment analysis for the comparison between AdiWT and AdiCent. **B** Heatmap visualization depicting the fold changes of the top 10 DEGs for the comparison between AdiWT and AdiCent. The color intensity represents the fold change in gene expression, with red indicating upregulation and blue indicating downregulation

As human centenarian associated SIRT6 N308K/A313S mutant trigger distinct epigenomic changes at the histone PTM level and this may impact gene expression, we sought to examine whether the transcriptional effects elicit by OE of SIRT6 WT or SIRT6 N308K/A313S in 3T3-L1 may differ before or after adipogenic differentiation, to gain functional insights additional to lipid accumulation. Three 3 T-3L1 cell groups were generated by LV-mediated infection: preadipocytes OE an empty vector (preAdiE), preadipocytes OE SIRT6 WT (preAdiWT) and preadipocytes OE SIRT6 N308K/A313S (preAdiCent, for “centenarians”). In turn, the three preadipocytes cell lines were differentiated into mature adipocytes: AdiE, AdiWT and AdiCent, respectively, and RNA-Seq was performed to assess the overall gene expression differences between the six groups (preAdiE, preAdiWT, preAdiCent, AdiE, AdiWT and AdiCent). Principal component analysis (PCA) was employed for exploratory analyses of the datasets onto two (Fig. 5A) or three dimensions (Fig. 5B), to capture the largest amount of variation, and revealed a neat clustering between the six groups. Next, we conducted comparisons between groups (preadipocytes *versus* preadipocytes, preadipocytes *versus* adipocytes, adipocytes *versus* adipocytes) using a p value of 0.055 and a threshold for |log2FoldChange| of 1.5. Considering the comparison between preAdiE versus preAdiCent identified 31 DEGs, of which 15 were up-regulated and 16 down-regulated (Figure S2A); comparing preAdiE v*ersus* preAdiWT, we identified 41 differentially expressed genes (DEGs), of which 21 were up-regulated and 20 down-regulated (Figure S2B); comparing preAdiWT *versus* preAdiCent identified the largest differences between preadipocyte groups with 57 DEGs, of which 36 were up-regulated and 21 down-regulated (Figure S2C). Interestingly, only 1 DEG was found in common between the three intra-preadipocytes comparisons (Figure S3A). Next, we compared preadipocytes *versus* the respective mature adipocyte cell lines, which highlighted larger SIRT6-dependent transcriptional changes: comparing preAdiE versus AdiE identified 450 DEGs, of which 216 were up-regulated and 234 down-regulated (Figure S4A, Supplemental File 1); comparing preAdiWT *versus* AdiWT identified 363 DEGs, of which 200 were up-regulated and 163 down-regulated (Figure S4B, Supplemental File 2); comparing preAdiCent *versus* AdiCent analysis identified 352 DEGs, of which 171 were up-regulated and 181 down-regulated (Figure S4C, Supplemental File 3). We found 244 DEGs in common between the three preadipocytes *versus* adipocytes comparisons: between these DEGs, ZBTB16, ACSL1, TIMP3, KLF15, NAMPTP1 and EMP1 were consistently differentially expressed between adipocytes and preadipocytes (Figure S3B). We then compared the transcriptional profiles among mature adipocytes. Comparing AdiE v*ersus* AdiWT analysis identified 85 DEGs, of which 27 were up-regulated and 58 down-regulated (Fig. 6A, Supplemental File 4); comparing AdiE *versus* AdiCent analysis identified analysis identified 58 DEGs, of which 37 were up-regulated and 21 down-regulated (Fig. 6B, Supplemental File 5); and comparing AdiWT *versus* AdiCent identified 102 DEGs, of which 64 were up-regulated and 38 down-regulated (Fig. 6B, Supplemental File 6). Only 3 DEGs were found in common between the three intra-adipocytes comparisons (Figure S3C). Beyond local lipid metabolism and accumulation, adipocytes substantially affect regulation of remote tissues through the endocrine action of adipokines and other mediators that in turn associate with widespread noncommunicable diseases, including cardiometabolic diseases, cancer and immune disorders [56]. Hence, the observed differences in transcriptional programs intra-preadipocytes groups and intra-adipocytes groups triggered by OE of SIRT6 (WT or Cent) led us to investigate in more depth differentially activated signaling pathways between AdiWT *versus* AdiCent mature adipocytes. An enrichment analysis using Ingenuity Pathway Analysis (IPA), applying a filter of both *p* value < 0.055 and abs(log2foldchange) > 1.5 and performed on the differential expression table for the AdiWT *versus* AdiCent comparison identified i. signaling by NTRK1 (TRKA) (represented genes: DNAL4, DNM1, DUSP7, FRS2, IRS1, IRS2, KIDINS220, PIK3CA), ii. PI3K cascade (represented genes: FRS2, IRS1, IRS2, PDE3B, PIK3CA), and iii) p53 signaling (represented genes: CCND2, CCNG1, PCNA, PIK3CA, THBS1, TP53BP2) as the top 3 differentially regulated canonical pathways (Fig. 7). H3K9ac and H3K14ac are associated with active promoters and are considered as hallmarks of active transcription [57]. As global H3K9ac and H3K14ac increased levels, among other acetylation marks, were detected in AdiCent compared to AdiE by mass-spec (Fig. 2), we sought to test whether differential acetylation may occur at the promoter regions of the genes identified by our IPA analysis. To this purpose, we considered three representative genes of the PI3K cascade (IRS1, IRS2, PIK3CA) and studied the acetylation level of H3K9/H3K14 to their promoter regions, using ChIP-qPCR experiments, in AdiCent compared to AdiE. Interestingly, a three- to fourfold increase in H3K9ac/H3K14ac was detected in AdiCent for both IRS1 and IRS2, while a 50% decrease was detected for PIK3CA (Supplementary Fig. 5), which may reflect their transcriptional status (Fig. 7). As negative controls, H3K9ac/H3K14ac occupancy of non-specific mouse genomic regions (Long interspersed nuclear element-1, LINE-1) and normal IgG were used (Supplementary Fig. 5).

### Overexpression of centenarian-associated (N308K/A313S) SIRT6 antagonizes neuropeptide Y (NPY) signaling and increases insulin sensitivity in mature adipocytes

**Fig. 8:**
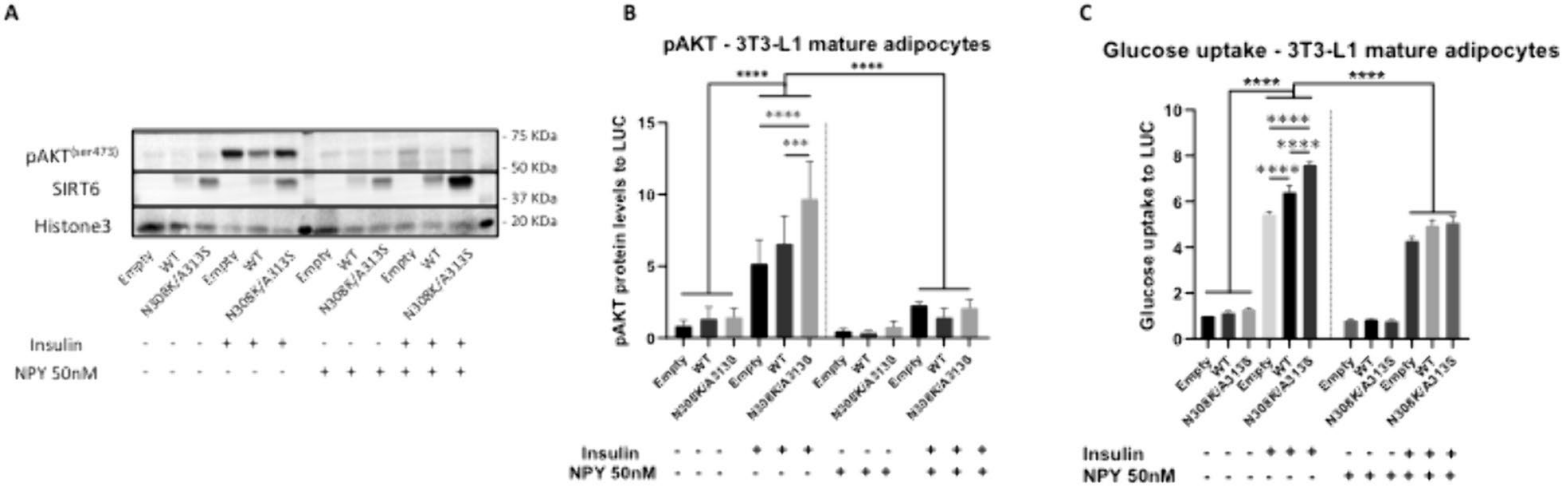
LV-mediated SIRT6 mutant overexpression and its impact on insulin sensitivity in 3T3-L1 cells. **A** immunoblotting to assess pAKT^Ser473^ and SIRT6 protein expression levels, in cells infected with LV-SIRT6 (WT or N308K/A318S) or empty control. **B** Densitometric quantification as in (**B**). (**C**) 3T3-L1 cells infected with LV-SIRT6 (WT or N308K/A318S) or empty control were treated with/without 50 nM NPY for 12 h and/or 100 nM insulin (INS) for 30 min to detect basal glucose uptake. ***p < 0.001, ****p < 0.0001 (Mann–Whitney *U* test)

Our transcriptomic analysis showed a major upregulation of cell cycle genes (PCNA, CCND2) and a significant downregulation of pro-apoptotic gene TP53BP2 (also known as apoptosis-stimulating of p53 protein 2 (ASPP2)) in AdiCent compared to AdiWT mature adipocytes (Fig. 7). RNA-Seq analysis also showed an enrichment of insulin signaling effector genes (IRS1, IRS2) and a depauperation of genes related to peripheral tissue innervation (FRS2, Fibroblast Growth Factor Receptor Substrate 2—enabling fibroblast growth factor receptor binding activity and neurotrophin TRKA receptor binding activity that in turn regulates innervation of the adipose tissue [58]; KIDINS220 – a downstream target of neuronal signaling events initiated by neurotrophins that plays a negative role in adipocyte maturation [59]; and DNM1—implicated in clathrin-mediated endocytosis at the synapse [60], in AdiCent compared to AdiWT mature adipocytes (Fig. 7). Neuropeptide Y (NPY) is one of the most common peptides in the brain and is an abundant neurotransmitter in the peripheral sympathetic nervous system. NPY has been shown to play a role in energy metabolism and obesity [61, 62]. Long et al*.* showed that in 3T3-L1 adipocytes treatment with NPY inhibited glucose uptake and decreased PI3K-AKT pathway signaling, suggesting NPY-dependent establishment of adipose tissue insulin resistance in mature adipocytes [63]. Interestingly, AdiCent were more sensitive to insulin stimulation compared to AdiWT or control AdiE mature adipocytes, as demonstrated by AKT phosphorylation (Fig. 8A, B) and glucose uptake (Fig. 8C). SIRT6 centenarian mutant-associated insulin hypersensitivity was blunted by 12 h preincubation with NPY (50 nM) (Fig. 8A–C), suggesting a potential involvement of cell signaling related to sympathetic nervous system innervation.

## Discussion

Adipogenesis is a possible therapeutic approach for fighting the obesity pandemic. Long-lived individuals, such as centenarians, display reduced rates of obesity compared to younger ones [24], and therefore, it is of interest to study their adipogenic mechanisms. Ashkenazi Jews centenarians carry SIRT6 variants (N308K and/or A313S) with enhanced mono-ADP-ribosylation activity [21]. In this work, we compared the transcriptional and epigenetic impact of SIRT6 N308K/A313A mutant versus SIRT6 WT during adipogenic differentiation in the lentivirus (LV)-infected 3T3-L1 in vitro model. The four main findings of our work were that overexpression of centenarian-associated SIRT6 mutant, compared to its WT counterpart: i) increased adipogenic differentiation to a similar extent; ii) triggered distinct histone PTM profiles in mature adipocytes, with significantly higher acetylation levels; iii) activated divergent transcriptional programs during adipocyte differentiation, including those dependent on signaling by NTRK1 and the PI3K cascade as most differentially regulated pathways; iv) increased insulin sensitivity in NPY-dependent manner.

First, our findings on adipogenic differentiation (i) mirror the fact that SIRT6 deficiency in 3T3-L1 preadipocytes blocks their adipogenesis [20]. Chen et al*.* showed that SIRT6 is essential for mitotic clonal expansion during adipogenesis [20], where growth-arrested preadipocytes synchronously re-enter the cell cycle and undergo several rounds of cell divisions. SIRT6 N308K/A313S and SIRT6 similarly increase the efficiency of adipocyte maturation in our LV 3T3-L1 model. Interestingly, our RNA-Seq data showed a major upregulation of cell cycle genes (PCNA, CCND2) and a significant downregulation of pro-apoptotic gene TP53BP2 in SIRT6 N308K/A313S-overexpressing mature adipocytes (Fig. 7): whereas mature adipocytes are post-mitotic and we could not detect gross differences in proliferation rates between the cell lines, we cannot exclude that SIRT6 N308K/A313S-overexpression may induce a small sub-population of mature adipocytes to resume proliferation, a captured phenomenon occurring in 3T3-L1 [64], whose physiological relevance in the context of obesity remains controversial [65]. Adipocyte differentiation is regulated by many pathways, among which histone acetylation plays a key role as one of the important epigenetic modifications [66]. Second, the main finding (ii) of our analysis of histone acetylation/methylation PTMs by mass spectrometry (LC–MS/MS) in 3T3-L1 differentiated adipocytes and uncovered a global histone hyperacetylation in mature adipocytes overexpressing SIRT6 N308K/A313S. This corroborates the biochemical assays of Simon et al*.*, showing that centSIRT6 displayed weaker deacetylase activity, but stronger mADPr activity, over a range of NAD + concentrations and substrates [21]. SIRT6 N308K/A313S overexpression has also been mechanistically linked to increased genome stability, including more efficient DNA double strand break repair and LINE1 retrotransposon suppression [21]. Clearly, obesity and increased adipose tissue depots have been associated with genome instability [2]. However, it appears that reduced genomic stability in obesity is not due to lipid accumulation per se, but it is the consequence of several factors that commonly accompany the condition, such as chronic inflammation, oxidative stress and altered insulin sensitivity/glucose uptake [67].

In this respect to the latter, our transcriptomic findings (iii) indicate that the significant upregulation of IRS1 and IRS2 in SIRT6 N308K/A313S-overexpressing mature adipocytes may be linked to the observed increase in glucose uptake, which in turn has been linked to the production of adiponectin in 3T3-L1 cells [68]. Moreover, we found a downregulation of genes related to adipose tissue innervation (FRS2, TRKA, KIDINS220, DNM1) [58–60], which led us to test (iv) the involvement of NPY, an abundant peripheral neurotransmitter, previously shown to inhibit glucose uptake and decrease PI3K-AKT pathway signaling in mature adipocytes [63]. Our data are consistent with these previous reports and indicate that the observed insulin hypersensitivity in SIRT6 N308K/A313S-overexpressing mature adipocytes may depend on decreased basal NPY signaling network, which in turn could be rescued by the supplementation of exogenous NPY to mature adipocytes. The role of SIRT6 overexpression in impairing sympathetic innervation signaling has been well documented in hypothalamic pro-opiomelanocortin (POMC)-expressing neurons [69]; however our work is the first report in adipocytes. Autonomic dysfunction in response to a high-fat diet contributes to adipose tissue expansion and recruitment of inflammatory cells: hypoxia, chronic inflammation and the overactivity of sympathetic nerves may be causing deregulation of NPY receptors [70, 71].

## Conclusions and future directions

In sum, our results reveal a comprehensive and specific epigenetic and transcriptomic profile in mature adipocytes upon overexpression of a SIRT6 mutant (N308K/A313S), which has a higher prevalence in healthy Ashkenazi centenarians. While the adipogenic process was seemingly unaffected, SIRT6 N308K/A313S overexpression in mature adipocytes led to an ameliorated glucose sensitivity and a deregulation of sympathetic innervation signaling. Our study presents with limitations. The in vitro deacetylase activity (acetyl group) of SIRT6 was shown to be hundreds-fold less potent than its long-chain defatty-acylase activity (malonyl, succinyl, butyryl, myristoyl and palmitoyl groups) on lysine residues [72]. These previously underappreciated histone PTMs are increasingly recognized molecular links between metabolism and epigenetic regulation of gene expression [73]; the role of centSIRT6 enzymatic activity on long-chain fatty acyl lysines remains to be analyzed. A further challenge is understanding how the increased mono-ADP ribosyl-transferase, as in SIRT6 N308K/A313S overexpression in mature adipocytes, may reflect in ameliorated glucose metabolism in the context of obesity [74–76]. A major limitation of our findings is that they are entirely based on an established in vitro model. If confirmed in vivo, for example through the generation of “humanized” mice models carrying N308K/A313S, these finding could highlight the importance of targeting SIRT6 pharmacology with compounds differentially targeting its multiple enzymatic activities to regulate the co-morbidities associated with obesity.

### Supplementary Information

Supplementary Material 1. Figure S1. Characterization of H3.1, H3.3 and H4 proteins in differentiated adipocytes using LC-MS/MS. Only highly confident peptides identified using a fixed-value PSM validator node with Mascot parameters set to Rank 1, expectation value < 0.01 and ion score ≥ 30 were considered. Sequence coverage (SC) is shaded in colour and numerically indicated in the right. Identified PTMs are indicated. Quantified peptides are marked in rectangles.

Supplementary Material 2. Figure S2. Volcano Plot visualization of Differentially Expressed Genes (DEGs) between pre-adipocytes . The x-axis represents the log2 fold change (log2FC), and the y-axis represents the -log10 of the p value. Genes with significant differential expression are highlighted in blue (up-regulated genes) and red (down-regulated) and are reported in the flanking table with the same color-code. (A) PreAdiE vs PreAdiCent. (B) PreAdiE vs PreAdiWT. (C) PreAdiWT vs PreAdiCent.

Supplementary Material 3. Figure S3. A. Venn diagrams illustrating the overlap of differentially expressed genes (DEGs) identified from the differential expression analysis across the preadipocytes and adipocytes comparisons: (A) Preadipocytes; (B) Adipocytes and Preadipocyte; (C) Adipocytes. Each circle represents a comparison, and the intersections represent genes that are commonly differentially expressed across the respective conditions. The numbers within each region indicate the count of shared DEGs.

Supplementary Material 4. Figure S4: Volcano Plot visualization of Differentially Expressed Genes (DEGs) between pre-adipocytes and adipocytes . The x-axis represents the log2 fold change (log2FC), and the y-axis represents the -log10 of the p value. Genes with significant differential expression are highlighted in blue (up-regulated genes), and red (down-regulated), and are reported in the flanking table with the same color-code. (A) AdiCent vs PreAdiCent. (B) AdiE vs PreAdiE. (C) AdiWT vs PreAdiWT.

Supplementary Material 5. Figure S5. Comparison of the occupancy of IRS1 (A), IRS2 (B), PIK3CA (C) promoters, and non specific genomic regions (LINE-1, D) by H3K9/K14Ac in AdiCent versus AdiE. Negative controls included normal IgG. Chromatin immunoprecipitation (ChIP)-quantitative PCR (qPCR) was performed with specific primers against the above mentioned genomic regions (A, B, C, D). Data represent the average of 3 biological replicates with error bars indicating SEM. P values comparing cell variants are indicated. Asterisks indicate statistically significant differences between AdiCent and AdiE. (*p < 0.05, ***p < 0.001).

Supplementary Material 6. Supplemental File 1: Differential expressed genes (DEG) in preAdiE versus AdiE.

Supplementary Material 7. Supplemental File 2: Differential expressed genes (DEG) in preAdiW versus AdiW.

Supplementary Material 8. Supplemental File 3: Differential expressed genes (DEG) in preAdiCent versus AdiCent.

Supplementary Material 9. Supplemental File 4: Differential expressed genes (DEG) in AdiE versus AdiW.

Supplementary Material 10. Supplemental File 5: Differential expressed genes (DEG) in AdiE versus AdiCent.

Supplementary Material 11. Supplemental File 6: Differential expressed genes (DEG) in AdiW versus AdiCent.

## Data Availability

The mass spectrometry proteomics data have been deposited to the ProteomeXchange Consortium via the PRIDE [72] partner repository with the dataset identifier PXD051761.
