# Supplementary figures and images for: Epigenetic and transcriptional control of adipocyte function by centenarian-associated SIRT6 N308K/A313S mutant
Source: Clin Epigenetics. 2024 Jul 20;16:96. doi: 10.1186/s13148-024-01710-1 (PMC11265064; doi:10.1186/s13148-024-01710-1)

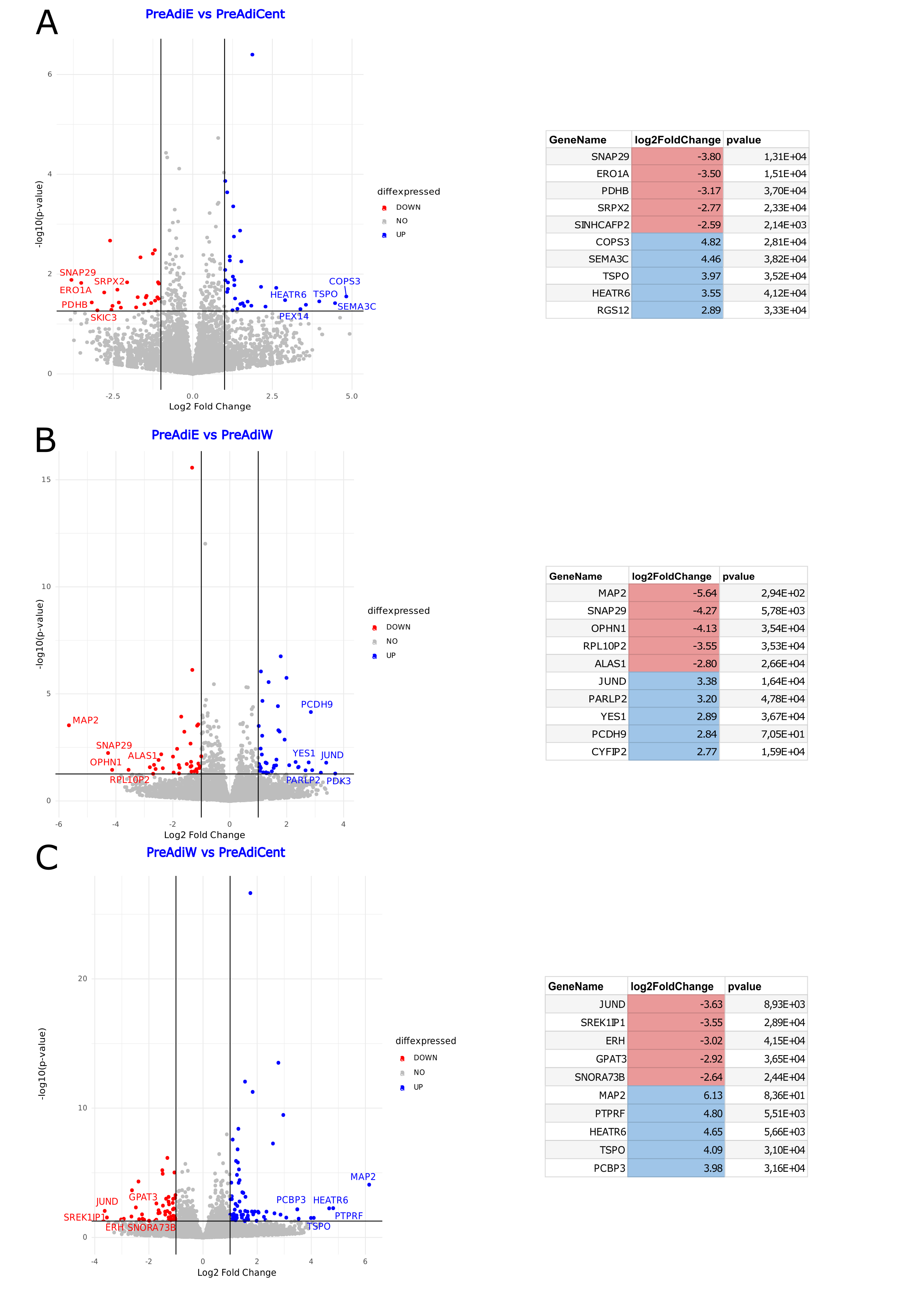

Supplement: Supplementary file 2 — Supplementary Material 2. Figure S2. Volcano Plot visualization of Differentially Expressed Genes (DEGs) between pre-adipocytes . The x-axis represents the log2 fold change (log2FC), and the y-axis represents the -log10 of the p value. Genes with significant differential expression are highlighted in blue (up-regulated genes) and red (down-regulated) and are reported in the flanking table with the same color-code. (A) PreAdiE vs PreAdiCent. (B) PreAdiE vs PreAdiWT. (C) PreAdiWT vs PreAdiCent. [file 13148_2024_1710_MOESM2_ESM.png]

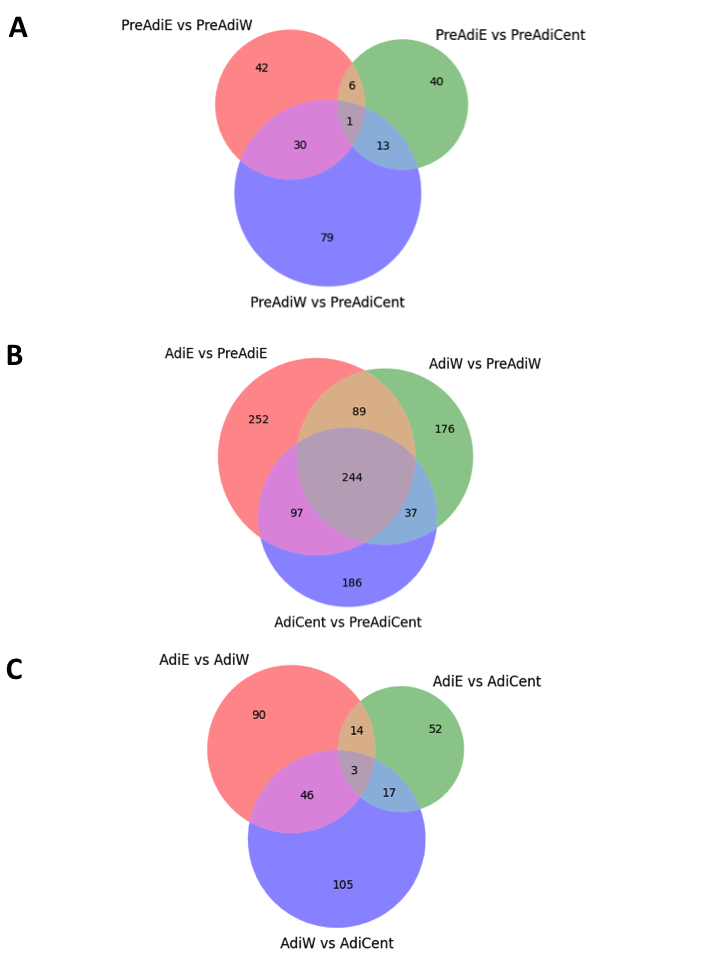

Supplement: Supplementary file 3 — Supplementary Material 3. Figure S3. A. Venn diagrams illustrating the overlap of differentially expressed genes (DEGs) identified from the differential expression analysis across the preadipocytes and adipocytes comparisons: (A) Preadipocytes; (B) Adipocytes and Preadipocyte; (C) Adipocytes. Each circle represents a comparison, and the intersections represent genes that are commonly differentially expressed across the respective conditions. The numbers within each region indicate the count of shared DEGs. [file 13148_2024_1710_MOESM3_ESM.tif]

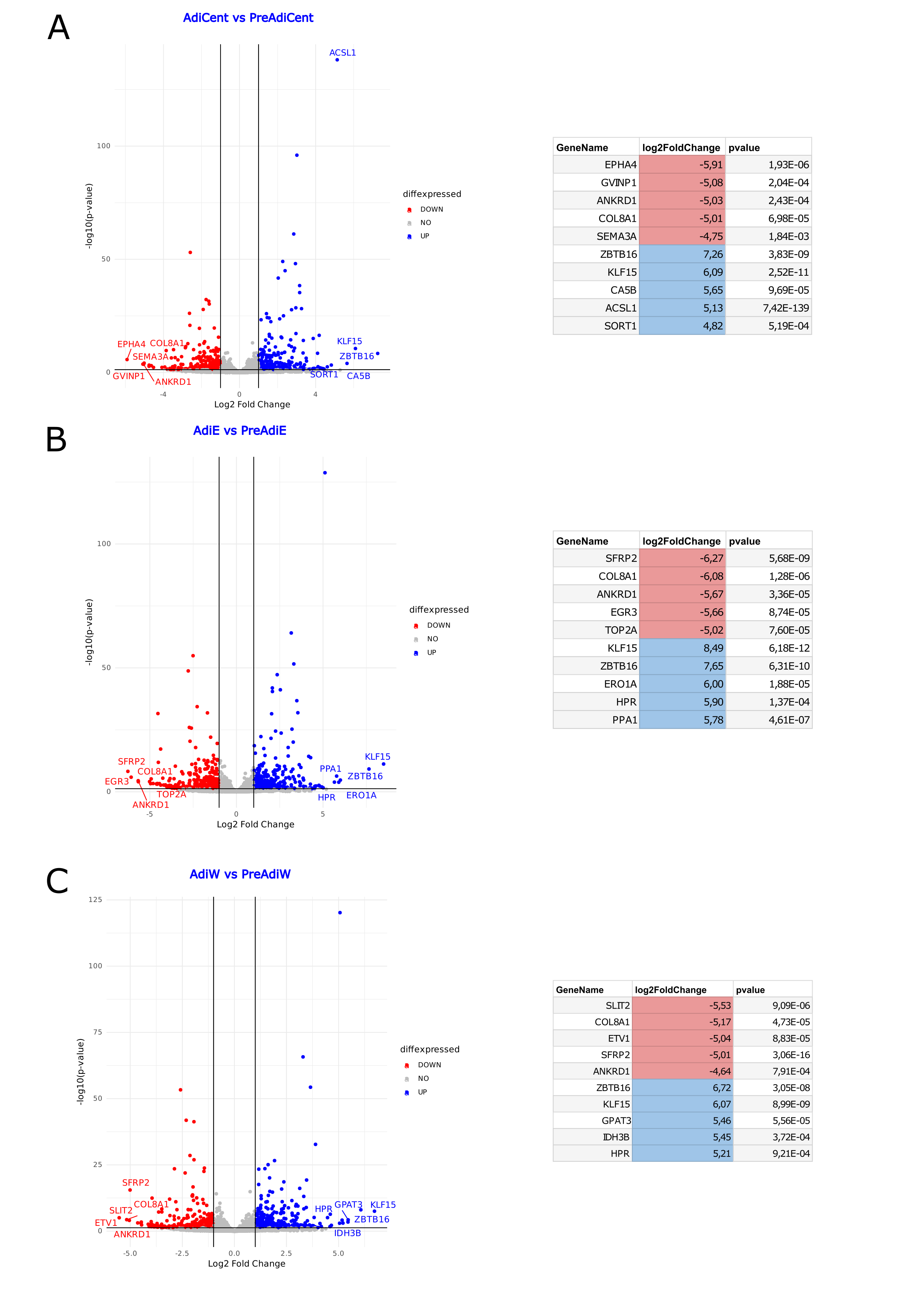

Supplement: Supplementary file 4 — Supplementary Material 4. Figure S4: Volcano Plot visualization of Differentially Expressed Genes (DEGs) between pre-adipocytes and adipocytes . The x-axis represents the log2 fold change (log2FC), and the y-axis represents the -log10 of the p value. Genes with significant differential expression are highlighted in blue (up-regulated genes), and red (down-regulated), and are reported in the flanking table with the same color-code. (A) AdiCent vs PreAdiCent. (B) AdiE vs PreAdiE. (C) AdiWT vs PreAdiWT. [file 13148_2024_1710_MOESM4_ESM.png]

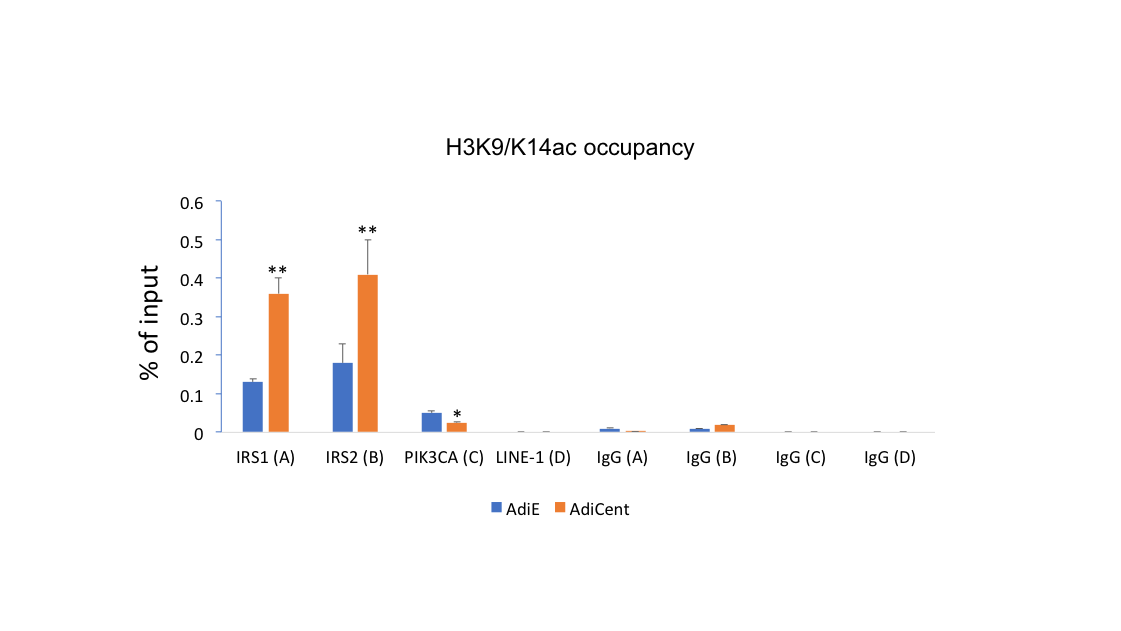

Supplement: Supplementary file 5 — Supplementary Material 5. Figure S5. Comparison of the occupancy of IRS1 (A), IRS2 (B), PIK3CA (C) promoters, and non specific genomic regions (LINE-1, D) by H3K9/K14Ac in AdiCent versus AdiE. Negative controls included normal IgG. Chromatin immunoprecipitation (ChIP)-quantitative PCR (qPCR) was performed with specific primers against the above mentioned genomic regions (A, B, C, D). Data represent the average of 3 biological replicates with error bars indicating SEM. P values comparing cell variants are indicated. Asterisks indicate statistically significant differences between AdiCent and AdiE. (*p < 0.05, ***p < 0.001). [file 13148_2024_1710_MOESM5_ESM.tif]
